# Screening criteria of mRNA indicators for wound age estimation

**DOI:** 10.1080/20961790.2021.1986770

**Published:** 2022-05-11

**Authors:** Qiuxiang Du, Tana Dong, Yuanxin Liu, Xiyan Zhu, Na Li, Lihong Dang, Jie Cao, Qianqian Jin, Junhong Sun

**Affiliations:** a School of Forensic Medicine, Shanxi Medical University, Jinzhong, China; b Shandong Public Security Department, The Institute of Criminal Science and Technology, Jinan, China; c Department of Military Traffic Medicine, Army Characteristic Medical Center, Chongqing, China

**Keywords:** Forensic sciences; forensic pathology, wound age estimation, mRNA indicator, ARE structure, GO categories

## Abstract

Wound age estimation is a crucial and challenging problem in forensic pathology. Although mRNA is the most commonly used indicator for wound age estimation, screening criteria are lacking. In the present study, the feasibility of screening criteria using mRNA to determine injury time based on the adenylate-uridylate-rich element (ARE) structure and gene ontology (GO) categories were evaluated. A total of 78 Sprague-Dawley male rats were contused and sampled at 4, 8, 12, 16, 20, 24, 28, 32, 36, 40, 44, and 48 h after inflicting injury. The candidate mRNAs were classified based on with or without ARE structure and GO category function. The mRNA expression levels were detected using qRT-PCR. In addition, the standard deviation (STD), mean deviation (MD), relative average deviation (d%), and coefficient of variation (CV) were calculated based on mRNA expression levels. The CV score (CVs) and the CV of CV (CV’CV) were calculated to measure heterogeneity. Finally, based on classic principles, the accuracy of combination of candidate mRNAs was assessed using discriminant analysis to construct a multivariate model for inferring wound age. The results of homogeneity evaluation of each group based on CVs were consistent with the MD, STD, d%, and CV results, indicating the credibility of the evaluation results based on CVs. The candidate mRNAs without ARE structure and classified as cellular component (CC) GO category (ARE–CC) had the highest CVs, showing the mRNAs with these characteristics are the most homogenous mRNAs and best suited for wound age estimation. The highest accuracy was 91.0% when the mRNAs without ARE structure were used to infer the wound age based on the discrimination model. The accuracy of mRNAs classified into CC or multiple function (MF) GO category was higher than mRNAs in the biological process (BP) category. In all subgroups, the accuracy of the composite identification model of mRNA composition without ARE structure and classified as CC was higher than other subgroups. The mRNAs without ARE structure and belonging to the CC GO category were more homogenous, showed higher accuracy for estimating wound age, and were appropriate for rat skeletal muscle wound age estimation.

Supplemental data for this article is available online at https://doi.org/10.1080/20961790.2021.1986770 .

## Introduction

Skeletal muscle wound is one of the most common type of injuries in forensic cases due to the features of skeletal muscle, wide distribution, and shallow location. Deducing skeletal muscle wound age accurately, especially the early wound age, is an important and challenging problem in forensic science. Currently, researchers mostly use one or two types of methods, including immunohistochemistry, ELISA, qRT-PCR, or Western blotting, to determine injury time [1–3]. qRT-PCR is probably the most common method to estimate early skeletal muscle wound age due to its objectivity compared with histological methods and Western blotting [4]. Currently, qRT-PCR has been used to explore miRNA biomarkers for important reactions of burned skin, and mRNA has been extensively studied in estimating postmortem interval (PMI) [5,6]. However, the use of qRT-PCR indices is often an initial and important step, although screening indicators for determining time of injury have not been established.

The ideal biomarker indicator for estimating wound age should have the ability of chronotropic change at different injury time points with a higher homogeneity at the same injury time point [7]. Based on earlier research on wound age estimation using mRNA expression, some mRNA expression levels, such as the mRNA encoding membranous FZD4, showed significant homogeneity. In contrast, other biomarkers showed significant heterogeneity due to different expression levels between individuals [8]. Furthermore, a theoretical framework to select individual indicators and weights that reflect their relative homogeneity and the heterogeneity of the overall wound healing process is needed.

The smaller the differences in mRNA expression between individuals, such as less heterogeneity or higher homogeneity, the more the mRNA expression of an individual could represent the overall expression trend, thus, the reliability of indicators used to predict wound age would be more important. Therefore, the selection of mRNA with higher homogeneity is crucial to guarantee the prediction accuracy of wound age. The steady state of mRNA expression levels depends on the equilibrium of its synthesis and degradation [9]. Sharova et al. [10] have conducted in-depth studies on the stability of RNA in yeast and different organs of mice and humans; results showed that RNA stability is critical for RNA expression [10–12]. Furthermore, in studies by Deng et al. [13], the stable accumulation of mRNA was affected by mRNA degradation. mRNA degradation pathways include the deadenylation-dependent mRNA decay, the adenylate-uridylate-rich element (ARE)-binding protein pathway, and nonsense-medicated decay [14]. In AREs, the cis-elements are localised on the 3′-UTR and occur in up to 5%–8% of human transcripts [15,16], thereby enhancing destabilisation of mRNA transcripts [17]. AREs can be categorised into three classes, Class I: continuous multiple AUUUA pentamer with nearby U-rich regions or U stretches; Class II: at least two consecutive UUAUUUA (U/A) (U/A) motifs in a U-rich region; Class III: abundant AU/U assembly without classic AUUUA motif [18–20]. AREs regulate mRNA stability by binding various specific binding proteins (AUBPs or ARE-BPs). AUF1, TTP, HuR, and some other AUBPs have been studied in detail regarding the RNA recognition motif, functional mechanism, and influence on downstream genes [15,17, 21–23]. The ARE often consists of the mRNA in inflammatory cytokines and growth factors mainly used to estimate skeletal muscle wound age [4,24].

Furthermore, based on homogeneity of different subcellular localisations, mRNA stability may be affected by mRNA function. Gene ontology (GO) describes the attributes of gene products, as well as classifies and analyses the interaction between the categories [25–27]. The GO functional categories contain cellular components (CCs), biological processes (BPs), and molecular functions (MFs). The CC describes the location of gene products in the cell, BP represents the molecular process and stress response of various stimuli at the molecular level, and MF embodies the original and essential MFs [28–31]. Therefore, hypothetically, mRNAs in the different GO categories will degrade at different rates and affect the steady state of mRNA expression levels.

Based on the above-mentioned theory, in the present study, 28 mRNAs in one of the three GO categories, and mRNAs with or without ARE structures with the chronotropic change at different injury time points were screened. Their homogeneity was analysed by measuring the relative amounts of mRNA at different time points using qPCR to construct a theoretical framework for selecting a variety of different mRNAs as ideal indicators for estimating wound age.

## Methods and materials

### Screening and classification of candidate genes

The RNA-seq data generated for this study can be found in the GSE171243 and were based on the raw next-generation sequencing data of contused skeletal muscle from our previous research (unpublished data). Genes with log2-fold change (FC; Injured/Control) >1 or < −1 and adjusted *P*  <  0.05 were considered significant and subjected to further downstream analysis.

The AREScore offered by German Cancer Research Centre (Deutsches Krebsforschungszentrum, DKFZ) and ARED developed by King Faisal Specialist Hospital & Research Centre were used to query and evaluate the AREs of mRNAs. GO functional categories were acquired using DAVIID Bioinformatics Resources 6.8 developed by the National Institute of Allergy and Infectious Diseases (NIAID) at NIH. The candidate genes were classified based on three criteria: with or without ARE structure, GO categories, and the combined subgroup (ARE − CC, ARE − BP, ARE − MF, ARE + CC, ARE + BP, and ARE + MF).

### Animal model and tissue preparation

All procedures were performed according to the Guiding Principles for the Care and Use of Laboratory Animals [32] and approved by the Institutional Animal Care and Use Committee of Shanxi Medical University of China (Batch number of rats: SCXK (Jin), 2009-0001). Animals were treated humanely in accordance with the principles of the Guidelines for Animal Experimentation established by our university. The Institutional Animal Care and Use Committee of Shanxi Medical University of China allowed laboratory personnel to conduct this study after attending and completing training on how to ethically use experimental animals.

A total of 78 Sprague-Dawley male rats, approximately 10–12 weeks old, weighing 250–300 g, were purchased from the Animal Centre of Shanxi Medical University. All animals were kept in stainless steel cages and provided rat chow and water *ad libitum*, under 12-h light/12-h dark cycles at 22 °C–24 °C and relative humidity of 40%–60%. All rats were randomly distributed into a control group (*n* =  6) or the 4-, 8-, 12-, 16-, 20-, 24-, 28-, 32-, 36-, 40-, 44-, and 48-h (*n*  =  6/group) contusion subgroups. After rats were anesthetised with diethylether, their right posterior limbs were shaved using a depilatory agent (Nair; Carter Wallace, New York, NY, USA). The contusion group rats were placed on a foam bed; a 100-g counterweight was dropped from a height of 200 cm (1.96 J) through a clear Lucite guide tube onto the thigh muscles of the right posterior limb [20].

Animals were maintained under 12-h light/12-h dark cycles with free access to food and water and sacrificed with a lethal dose of pentobarbital (350 mg/kg body weight, intraperitoneal injection) at the predetermined time points. The rats in the control subgroup without contusion were sacrificed in the same manner. Near the right posterior limbs at the adductor longus muscle and gracilis muscle, approximately 100 mg of skeletal muscles was dissected and quick-frozen immediately with liquid nitrogen and stored until used for subsequent experiments.

### qPCR to detect mRNA expression level

Total RNA was extracted from skeletal muscle specimens (approximately 50-mg samples) using RNAiso Plus 9108 (Takara Bio Inc., Shiga, Japan) following the manufacturer’s instructions. The concentration (ng/mL) and purity of freshly extracted total RNA were evaluated using the Infinite microplate reader (M200 Pro; TECAN; Männedorf, Switzerland), and the integrity of total RNA was tested using Agilent 2100 (Agilent Technologies, Santa Clara, CA, USA) with the Agilent RNA 6000 Nano kit. Only RNAs with OD260/OD280 ratios of 1.8–2.2 and RNA integrity number (RIN) >7.0 that met the experimental conditions regarding the quality control of RNA were used.

According to the standard protocol, the synthesis of template cDNA was performed using the Prime Script RT-PCR Kit (Takara Bio Inc.). Each 10 mL reverse transcription reaction mix contained 500 ng total RNA.

Primers and TaqMan fluorescent probes were designed using the AlleleID 6 software (Premier Biosoft International, Palo Alto, CA, USA), examined with BLAST, and synthesized by Sangon Biotech (Shanghai, China). RPL13 mRNA and RPL32 mRNA were chosen as the reference mRNAs due to their stability in contused skeletal muscles as confirmed in our previous study [33]. The gene primers, probe sequences, and fluorescent labeling data are shown in Supplementary Table S1. Furthermore, to avoid the amplification of genomic DNA, the sense primers and anti-sense primers were designed to span genomic DNA introns.

The two reaction mixtures, which minimally differed in composition, were prepared for qPCR amplification using the Premix Ex Taq Kit (Takara Biotechnology Co. Ltd., Dalian, China); each mixture contained primers and probes for four genes including two reference genes and two target genes. One mixture contained 12.5 μL Premix Ex Taq, 1.5 μL RNase-free H_2_O, 2.0 μL cDNA, and 0.75 μL each of the TaqMan fluorescent probes and forward and reverse primers of a gene of interest (GOI) and the two reference genes. The other mixture included 12.5 μL Premix Ex Taq, 2.0 μL 10% DMSO, 1.2 μL RNase-free H_2_O, 1.5 μL cDNA, and 0.65 μL each of the primers and probes. The concentrations of the primers and probes are listed in Supplementary Table S1. Amplification was performed using Bio-Rad Real-time PCR System (CFX384; Bio-Rad, Hercules, CA, USA). The amplification process consisted of three different annealing and extension temperatures, 58 °C, 59 °C, and 60 °C, with one round of pre-denaturation at 95 °C for 10 s, and 40 rounds of denaturation at 95 °C for 5 s followed by annealing and extension for 40 s. Four fluorescent signals (FAM, Cy5, HEX, and ROX) were simultaneously recorded at the end of each cycle. Fluorescence curves of the PCR products were evaluated using Bio-Rad Real-time PCR System software. To detect the amplification efficiency, EASY Dilution solutions (Takara Bio Inc.) were used to dilute the synthesized cDNAs serially five-fold (1, 1:5^1^, 1:5^2^, 1:5^3^, and 1:5^4^). Deionized H_2_O was used as the negative control. All experiments were repeated in triplicate.

### Statistical analyses

The target mRNA expression levels were computed using the statistical model of (1 + Eff.)^−ΔΔCt^. Based on the relative expressions, mean deviation (MD), standard deviation (STD), relative average deviation (d%), and the coefficient of variation (CV) of each contusion group were calculated.

SPSS Version 24.0 (IBM Corp., Armonk, NY, USA) and R Studio 1.1.463 (https://www.npackd.org/p/rstudio/1.1.463) were used for statistical analysis. First, the CV score (CVs) and the CV of the CV (CV’CV) were calculated to measure the homogeneity of groups with the above-listed three classification criteria. The mRNA indicators were scored based on the order of CV in each wound age and the calculated sum of each indicator score in the contusion groups. The minimum 25% CV was assigned 4 points, 25%–50% CV was assigned 3 points, 50%–75% was assigned 2 points, and the maximum 25% CV was assigned 1 point because smaller CV represents greater homogeneity. Ultimately, the mRNAs with a higher score showed higher homogeneity. In addition, the CV was used as the original data and the CV of each indicator CV was computed (CV’CV).

To further test the results, Fisher discriminant analysis was used to build a multivariate model for inferring wound age based on the expression levels of mRNAs grouped into with or without ARE structure, GO categories, and the subgroup combined using the above-described classifications. All variables were entered together; Wilks’ λ and the cross-validation were used as the test statistic. *P* < 0.05 was considered to indicate statistical significance.

## Results

### Candidate mRNAs in different categories

Based on gene sequences, the mRNAs with an ARE structure on the ARED website and an ARE score >3 were denoted as with ARE structure (ARE+) groups. The mRNAs without ARE structure on the ARED website were denoted as without ARE structure (ARE−) groups. The details are presented in Supplementary Table S2. Subsequently, the mRNAs which were classified into two or three GO functional categories were excluded. The mRNAs that were classified into only one GO functional category were chosen and subdivided into the CC, BP, and MF subgroups. Finally, 28 mRNAs were selected as candidates for this study and the corresponding genes were divided as ARE − CC, ARE − BP, ARE − MF, ARE + CC, ARE + BP, and ARE + MF subgroups (Table 1).

**Table 1.** Genetic information of screening indicators.

Subgroup	Gene symbol and gene ID
ARE − CC	Rae1 (NM_001033708), Myg1 (NM_001005545), Rabepk (NM_001024871), Tmem100 (NM_001017479), Lin37 (NM_001106245), Lrrc41 (NM_001009710)
ARE − BP	Prrx2 (NM_001105739), Abhd2 (NM_001106275), Prr5 (NM_001012121), Fbxw4 (NM_001107600), Rhbdd3 (NM_001013875)
ARE − MF	Ipo4 (NM_001106038), Prr3 (NM_212544), Arid5a (NM_001034934), Trit1 (NM_001108676), Rcc1l (NM_001108332)
ARE + CC	Sc65 (NM_021581), Tmem45b (NM_001033067), Leprot (NM_020099), Fam210a (NM_001007688)
ARE + BP	Impact (NM_001012235), Asb5 (NM_001044247), Hs6st1 (NM_001108210), Ier3 (NM_212505)
ARE + MF	Samd4b (NM_001107498), Dclre1b (NM_001025687), Dennd5a (NM_001107546), Slfn3/4 (NM_0536870808)

ARE, adenylate-uridylate-rich element; ARE+, mRNAs with ARE structure; ARE−, mRNAs without ARE structure; CC, mRNAs classified as cellular componentcategory; BP, mRNAs classified as biological process category; MF, mRNAs classified as multiple function category

### The expression levels, MD, and STD of mRNAs in contused skeletal muscle

All total RNA OD260/OD280 ratios ranged from 1.8 to 2.0, indicating highly pure RNA. In addition, the RIN scores of total RNA samples were >7.0, indicating the quality of RNA met the requirements for follow-up experiments.

The amplification efficiencies of mRNAs, at least the mean of three repeats after the experimental conditions, were confirmed (Supplementary Table S2). All R squared (Rsq) values of mRNAs were >0.990, indicating accurate amplification efficiencies and appropriate experimental conditions.

**Figure 1.**
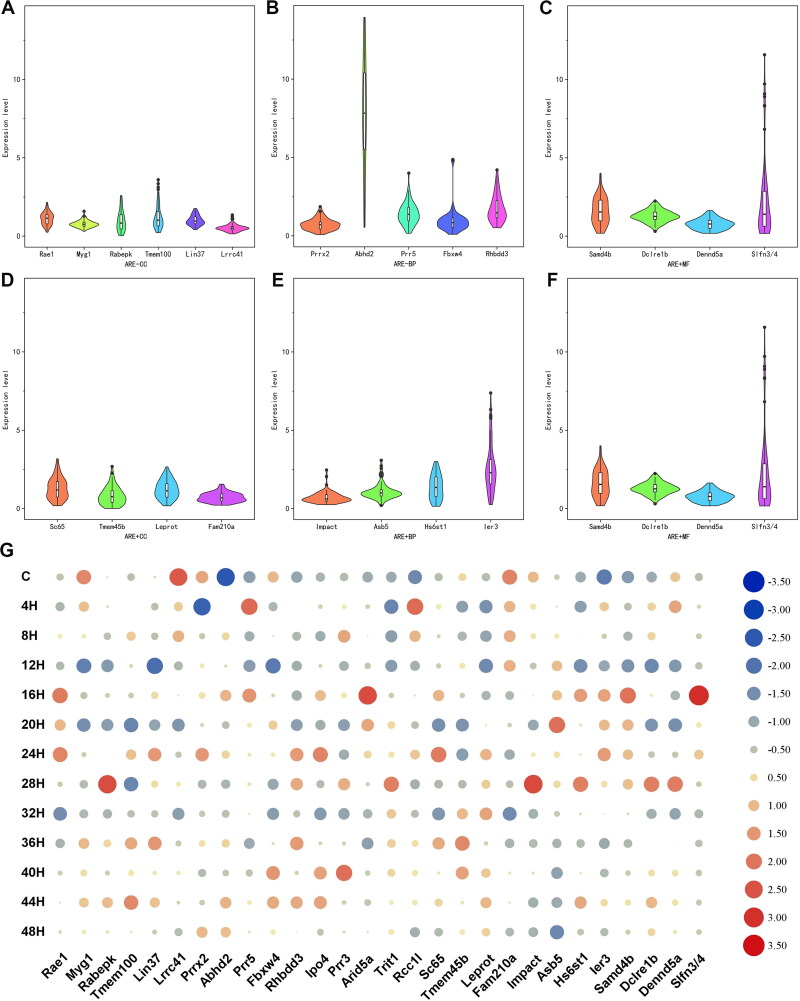
The expression changes of each mRNA. (A–F) were violin plots with mRNA expression levels of ARE − CC, ARE − BP, ARE − MF, ARE + CC, ARE + BP, and ARE + MF subgroups, respectively. The shape of the violin represents the distribution of the samples. The shorter and fatter the violin is, the more concentrated the distribution; the taller and thinner the violin is, the more dispersed the distribution. (G) Heat maps of mRNA expression changes. The value in the heat map is its standardisation according to its own expression level.

To further verify the sensitivity of the candidate mRNAs for wound age estimation, the mRNA expression levels were calculated using the statistical model (1 + Eff.) ^-ΔΔCt^. The statistical differences were analysed using one-way analysis of variance (ANOVA) and Student-Newman-Keuls methods. As shown in Figure 1 and Supplementary Table S2, all mRNA expression levels changed significantly at some time point. Each mRNA expression levels showed a specific time-dependent changing pattern (Figure 1G). For each mRNA indicator, the lower the range of its expression level, the higher the homogeneity. Similarly, the shorter the index in the violin chart, the smaller the individual differences and the higher the homogeneity. In conclusion, mRNA homogeneity was higher in the CC group.

**Figure 2.**
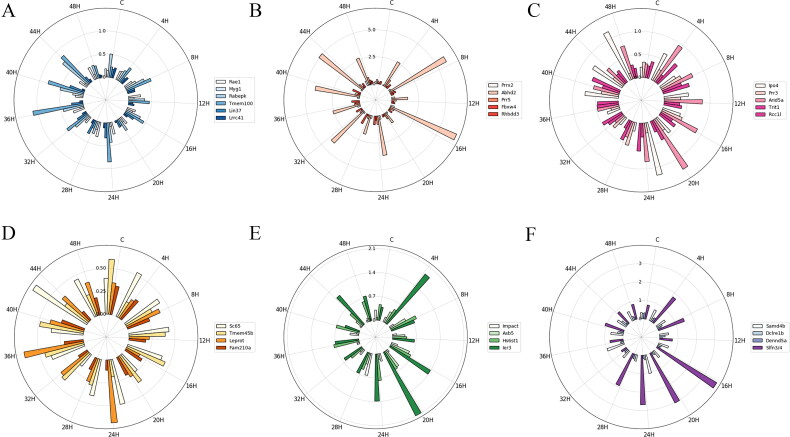
The mean deviation (MD) of mRNA expression in each subgroup at each injury time point. (A) ARE − CC; (B) ARE − BP; (C) ARE − MF; (D) ARE + CC; (E) ARE + BP; (F) ARE + MF. Each circle represents a numerical scale and the radial dividing line is used to distinguish the time of injury. The column diagram inside the circle shows the MD of mRNA expression at the injury time point and different colours represent different mRNAs. ARE, adenylate-uridylate-rich element; ARE+, mRNAs with ARE structure; ARE−, mRNAs without ARE structure; CC, mRNAs classified as cellular componentcategory; BP, mRNAs classified as biological process category; MF, mRNAs classified as multiple function category; C, control.

**Figure 3.**
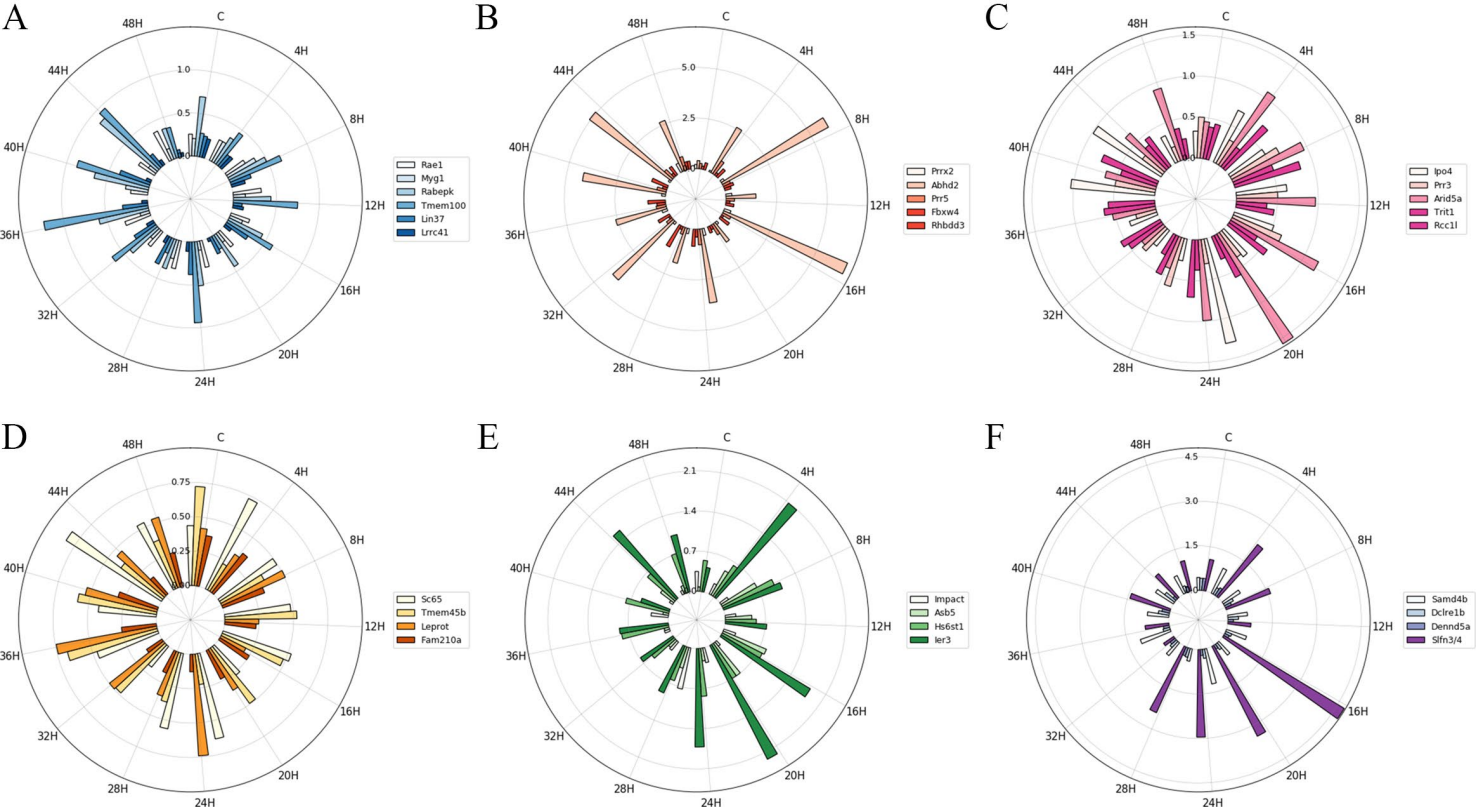
The standard deviation (STD) of mRNA expression in each subgroup at each injury time point. (A) ARE − CC; (B) ARE − BP; (C) ARE − MF; (D) ARE + CC; (E) ARE + BP; (F) ARE + MF. Each circle represents a numerical scale and the radial dividing line is used to distinguish the time of injury. The column diagram inside the circle shows the STD of mRNA expression at the injury time point and different colours represent different mRNAs. ARE, adenylate-uridylate-rich element; ARE+, mRNAs with ARE structure; ARE−, mRNAs without ARE structure; CC, mRNAs classified as cellular componentcategory; BP, mRNAs classified as biological process category; MF, mRNAs classified as multiple function category; C, control.

To understand the homogeneity of the candidate mRNAs categorised based on the structure and GO function in the control and the contusion groups, the MD and STD of each group were calculated to measure the variation degree based on the relative mRNA expression level. Figures 2 and 3 show that the MD and STD of mRNA in each subgroup indicated different temporal changes and that their radial histograms were similar.

### Homogeneity assessment of subgroup based on d% and CV

MD and STD are considered absolute differences. However, due to the differences in mRNA expression levels in each group, MD and STD could not be used to evaluate the homogeneity of indicators. Therefore, d% (d% = MD/mean × 100%) and CV (CV = STD/mean × 100%) were introduced as relative differences to evaluate the homogeneity of each mRNA.

**Figure 4.**
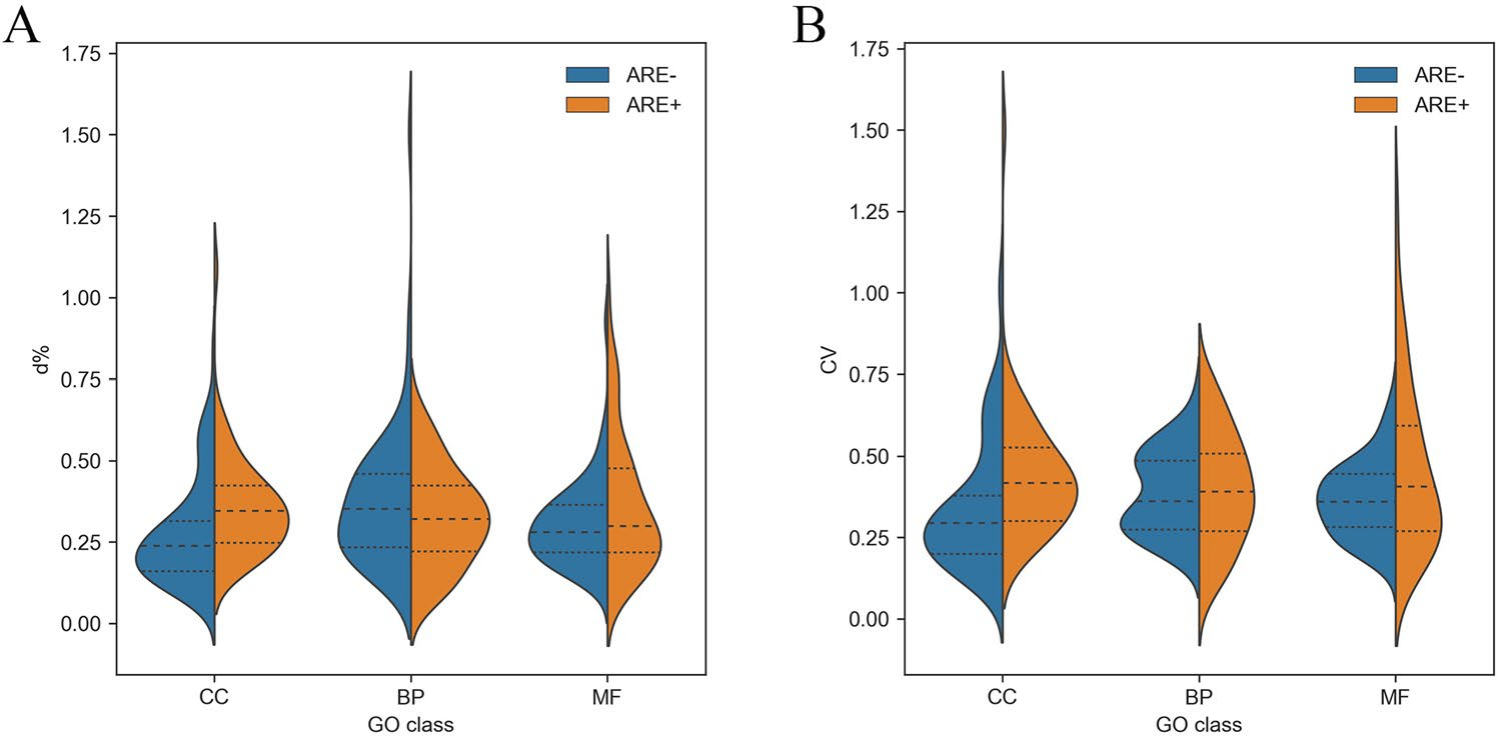
The d% (A) and CV (B) of mRNA expression level in each subgroup. Dotted lines indicate quartiles. The shape of the violin represents the distribution of the samples. The shorter and fatter the violin is, the more concentrated the distribution; the taller and thinner the violin is, the more dispersed the distribution. d%, relative average deviation; CV, coefficient of variation; GO, gene ontology; ARE, adenylate-uridylate-rich element; CC, mRNAs classified as cellular componentcategory; BP, mRNAs classified as biological process category; MF, mRNAs classified as multiple function category.

The d% of mRNA expression level in each subgroup was similar to CV. As shown in Figure 4A, among all subgroups, the mean d% and CV values in the ARE − CC subgroup were the lowest, indicating that mRNA homogeneity was the highest in this group. In addition, a statistically significant difference was observed between the ARE − CC and ARE + CC groups. Furthermore, CV results showed the homogeneity of mRNAs without ARE structure in each GO group was higher than mRNAs with ARE structure (Figure 4B).

### Homogeneity evaluated in subgroups based on CVs and CV’CV

CV scores represent the degree of homogeneity of all indicators at the same injury time point, and CV’CV represents the homogeneity of the same indicator at different injury time points. The CV scores and CV’CVs are shown in Supplementary Tables S3 and S4.

**Table 3.** The Fisher discrimination analysis for estimating injury time.

	Wilks’ *λ*	*P*-value				Accuracy (%)
ARE+structure	0.001	0.000				76.9
ARE−structure	0.000	0.000				91.0
CC	0.004	0.000				78.2
BP	0.021	0.000				61.5
MF	0.007	0.000				76.9
ARE−CC	0.027	0.000				66.7
ARE−BP	0.210	0.000				37.2
ARE–MF	0.050	0.000				61.5
ARE+CC	0.122	0.000				38.5
ARE+BP	0.197	0.000				34.6
ARE+MF	0.149	0.000				37.2

Note: The accuracy in the table is across validation accuracy. ARE, adenylate-uridylate-rich element; ARE+, mRNAs with ARE structure; ARE−, mRNAs without ARE structure; CC, mRNAs classified as cellular componentcategory; BP, mRNAs classified as biological process category; MF, mRNAs classified as multiple function category.

**Figure 5.**
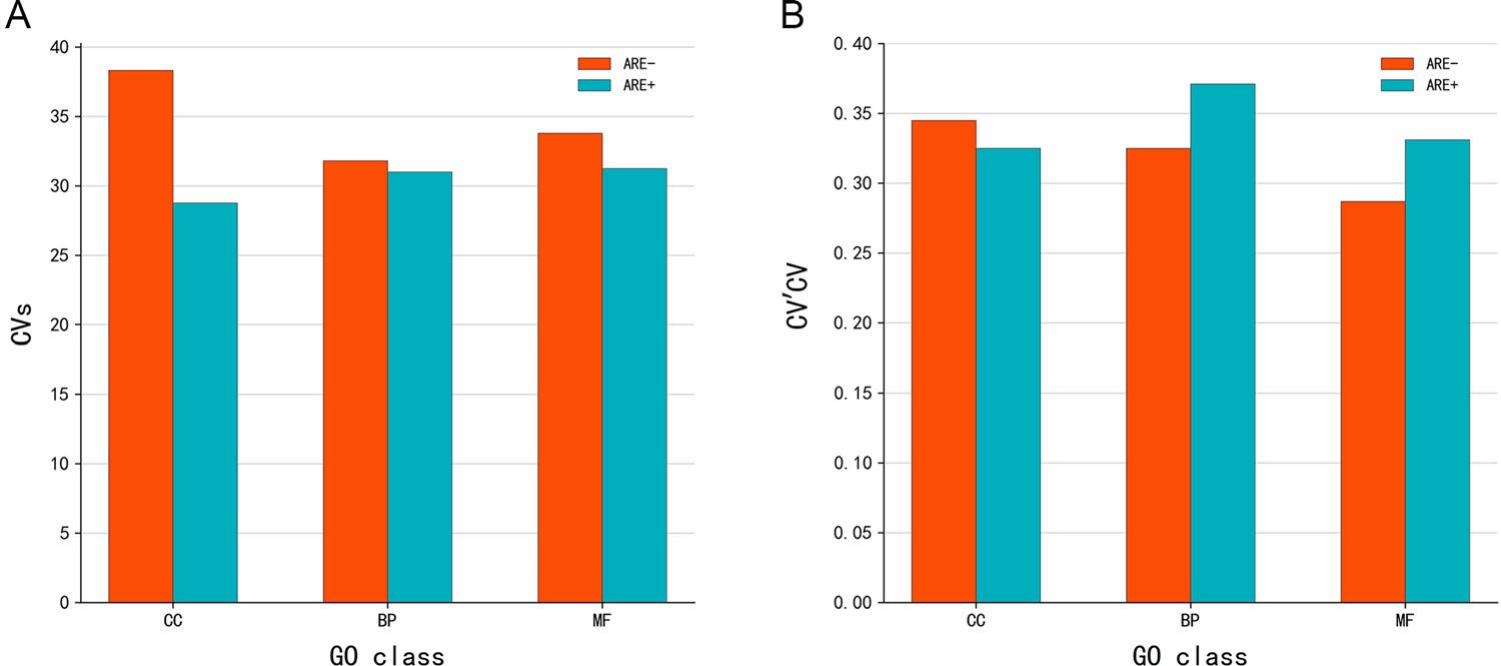
CVs (A) and CV’CV (B) of mRNA expression level in each subgroup. CVs, CV score; CV’CV, the CV of CV; GO, gene ontology; ARE, adenylate-uridylate-rich element; CC, mRNAs classified as cellular componentcategory; BP, mRNAs classified as biological process category; MF, mRNAs classified as multiple function category.

The CV scores of all candidate mRNAs which belong to each subgroup were calculated. The results in Figure 5A show the ARE − structure groups had higher CVs than ARE + structure groups. The different CVs based on GO categories ranged from high to low in CC, MF, and BP, in that order. The combination of the two classifications showed the ARE − CC subgroup had the highest CVs and ARE + CC subgroup the lowest CVs.

Similarly, the CV’CV of all candidate mRNAs in each subgroup were also calculated. As presented in Figure 5B, the ARE − structure group CV’CV value was 0.321 and the ARE + structure group was 0.343. Different CV’CV values based on MF, CC, and BP GO categories were observed. The CV’CV of ARE − MF, ARE − BP, ARE + CC, ARE + MF, ARE − CC, and ARE + BP were 0.287, 0.325, 0.325, 0.336, 0.345, and 0.371, respectively. The results showed that mRNAs without ARE structure differ less among individuals and have high homogeneity.

### Accuracy assessment of wound age estimation using Fisher discrimination analysis

Based on the calculation results of different subgroups, mRNA homogeneity was the highest in the ARE − CC group, followed by ARE − MF (Table 2). However, whether the mRNA with high expression homogeneity can be used to infer injury time in individuals with higher accuracy than other methods remains to be determined.

**Table 2.** Comprehensive evaluation of mRNA homogeneity in each subgroup.

	ARE − CC	ARE − BP	ARE − MF	ARE + CC	ARE + BP	ARE + MF
MD	0.267	0.969	0.464	0.336	0.511	0.640
STD	0.327	1.122	0.562	0.414	0.622	0.775
d%	0.279	0.374	0.304	0.358	0.356	0.326
CV	0.344	0.387	0.367	0.441	0.398	0.454
CV’CV	0.345	0.325	0.287	0.325	0.371	0.336
CVs	38.33	31.80	33.80	28.75	31.00	31.25

ARE–CC, mRNAs without ARE structure and classified as cellular component category; ARE–BP, mRNAs without ARE structure and classified as biological process category; ARE–MF, mRNAs without ARE structure and classified as multiple function category; ARE+CC, mRNAs with ARE structure and classified as cellular component category; ARE+BP, mRNAs with ARE structure and classified as biological process category; ARE+MF, mRNAs with ARE structure and classified as multiple function category; MD, mean deviation; STD, standard deviation; d%, relative average deviation; CV, coefficient of variation; CV’CV, the CV of CV; CVs, CV score.

**Figure 6.**
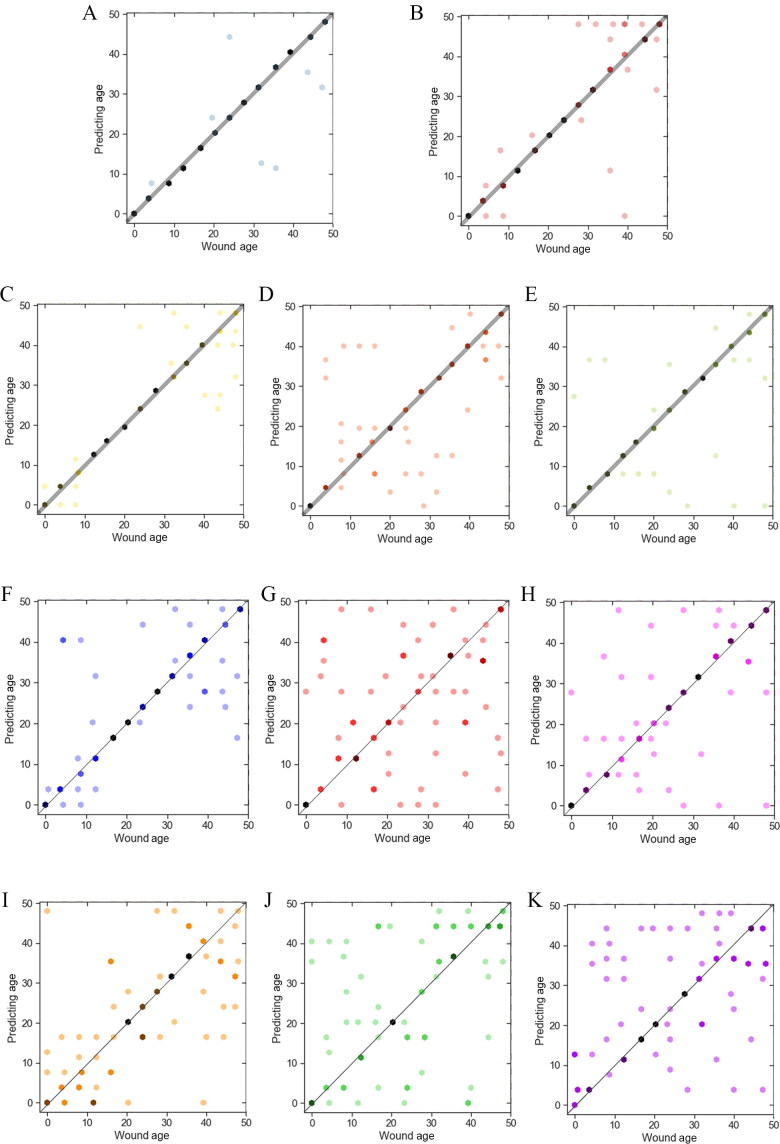
Fisher discriminant analysis of each group and each subgroup. One dot represents one or more sample. The more points that fall in the same position, the darker the colour. The points on the line are the samples with the correct prediction. (A) ARE − group; (B) ARE + group; (C) CC group; (D) BP group; (E) MF group; (F) ARE − CC; (G) ARE − BP; (H) ARE − MF; (I) ARE + CC; (J) ARE + BP; (K) ARE + MF. ARE, adenylate-uridylate-rich element; ARE+, mRNAs with ARE structure; ARE−, mRNAs without ARE structure; CC, mRNAs classified as cellular componentcategory; BP, mRNAs classified as biological process category; MF, mRNAs classified as multiple function category.

Therefore, the raw data of combination candidate mRNAs in different subgroups were entered into the Fisher discriminant analysis to determine the time of wound injury and the accuracy calculated. As shown in Figure 6 and Table 3, significant differences were observed between each subgroup. The discriminant functions were statistically significant because the Wilks’ *λ* values were almost always <0.5 with all *P*-values <0.05. Thus, mRNAs without ARE structure and classified into CC or MF GO categories were more accurate for predicting wound age. Combination of the above two methods showed the wound age estimation accuracy of ARE − CC subgroup had the highest accuracy, and ARE − MF had higher accuracy than ARE + CC, ARE + MF, ARE − BP, and ARE + BP subgroups.

## Discussion

Wound age estimation is always a crucial and challenging task in forensic pathology, and the early wound age estimation is an important problem to be solved urgently. mRNA expression took precedence over protein and tissue levels, making it more useful for early wound age estimation, although it is susceptible to other factors such as PMI, environmental impact and the circumstances of death. qRT-PCR is a standard method that is stable and objective and widely used in the life sciences. Due to the lack of mRNA screening criteria, the indicators and results used in each laboratory are different. In addition, different mRNA expression patterns showed significant individual differences, which also limits its application in practice. To avoid the inference error caused by individual differences, indices with minor individual differences can be used to determine wound age. In the present study, the mRNA structure and GO categories were used to analyse homogeneity of mRNA expression among different individuals and then determine the mRNA indicators suitable for wound age estimation.

In the theoretical framework of selecting the mRNA indicators, several algorithms should be used to determine the differential expression of different mRNA groups. The common statistical methods include fold change, *t*-test, and SAM, among others. The mRNAs selected using these methods can only represent differences in expression among different groups (inter-group differences). Having the between-group error sufficiently large and the within-group error sufficiently small is the basic principle to improve the accuracy of discrimination. In the present study, when selecting different mRNAs using traditional methods, MD, STD, d%, CV, CV’CV, and CVs were used to analyse the different mRNAs in each group to further optimise the screening procedure and render the selected indices more representative.

Within-group error is the random error from the data within the sample, which reflects the degree of difference within the sample data as well as between different samples. Individual difference is one cause of within-group error. After injury, the individual differences of each mRNA also differed. MD, STD, d%, and CV are commonly used to evaluate inter-individual heterogeneity [8]. Based on traditional evaluation of individual differences, CVs and CV’CV were used in the present study to evaluate mRNAs in each group, further eliminating the inaccuracy of evaluation caused by differences in mRNA expression between individuals; the results were consistent with MD, STD, d%, and CV results. Under the premise of a higher CVs, smaller CV’CV represents better homogeneity.

Previous study found that some genes made little contribution to wound age estimation, which decreased the accuracy of the model comprising control and 4–24 h and 28–48 h wound age groups; The accuracy of 14 of the 35 mRNAs in predicting wound age was similar to that of 35 mRNAs [34]. Nine of the 14 mRNAs used for wound age estimation were without ARE structure, and most of 14 mRNAs belonged to the CC and MF GO category and only three mRNAs were in the BP category; Among the nine mRNAs without ARE structure, four belonged to the ARE–CC subgroup (44.4%) and four belonged to the ARE–MF subgroup. In the present study, the above indicators were also used to evaluate the presence of ARE structure and mRNAs in different GO categories to explore whether structural and biological function differences influence mRNA homogeneity. As shown in the results, the accuracies of wound age inference in the ARE–CC subgroup (66.7%) and ARE–MF subgroup (61.5%) were higher (Table 3). The mRNAs of the ARE–CC subgroup had the lowest MD, STD, d%, and CV, and the highest CVs (Table 2), indicating the highest homogeneity.

The CV’CV value of mRNAs without ARE structure was lower than mRNAs with ARE structure, and the CVs of mRNAs without ARE structure was higher than mRNAs with ARE structure. The results of Fisher discriminant analysis showed the groups with the highest accuracy at different grouping levels were ARE– group (91.0%), CC group (78.2%), and ARE–CC subgroup (66.7%), respectively (Table 3). In this study, the injury time was strictly determined based on experimental design, and there was no merging of damage time points or stratified discrimination to improve accuracy [35]. The results showed that using the selected indices to determine the injury time ensures the sensitivity of prediction, simplifies the process of inference, and thus accuracy is improved.

Collectively, the results indicate the homogeneity of mRNAs satisfying the conditions mentioned above may be the highest, which is more suitable for predicting wound age. Furthermore, the mRNAs in the BP GO category had higher MD, STD, d%, and CV’CV and the lowest CVs compared with other categories, indicating the BP categories with or without ARE structure may be more heterogeneous.

In this study, all 28 mRNAs played a role in the injury and repair of skeletal muscle. In adult tissues, Myg1 is basically regulated in response for stress such as cytotoxicity, starvation and degeneration; its decrease occurs *in vitro* culture of C2C12 myoblast differentiation, and in some distribution of immune function deceases, its exerts up-regulation [36,37]. Slfn3/4 (schlafen 3/4) is the member of slfn family, which expressed in T cells, NK cells, monocytes, and macrophages, increased with the activation and proliferation of naive CD4 T cells and decreased during macrophages differentiation [38]. Ier3 participates in several survival pathways such as NF-κB, Akt and Erk, also named as immediate early gene X-1 (IEX-1), which mainly acts as the activation of myogenic progenitor cells enters cell cycle [39]. Myg1 had low MD and STD and belonged to the ARE–CC subgroup, while Slfn3/4 and Ier3 had higher MD and STD and were divided into ARE + MF and ARE + BP subgroups, respectively (Figures 2 and 3). The mRNAs that were without ARE structure and classified into CC GO category had less individual differences and higher homogeneity in high probability, and these mRNAs can more accurately estimate wound age.

In conclusion, the above results indicate that mRNAs without ARE structure and classified into CC or MF GO category should be given priority when screening indicators for predicting the time of skeletal muscle injury. However, the number of mRNAs involved in this study may not be large enough and the transformation of results from animal experiments to human applications may be a long way. We will continue to collect relevant information and use human samples to verify the results of this study in subsequent experiments, which could be helpful for screening indicators in future wound age studies.

## Supplementary Material

Supplemental Material
