# Supplementary material for: Screening criteria of mRNA indicators for wound age estimation
Source: Forensic Sci Res. 2022 May 11;7(4):714–25. doi: 10.1080/20961790.2021.1986770 (PMC9930757; doi:10.1080/20961790.2021.1986770)
Supplement: Supplemental Material [file TFSR_A_1986770_SM7044.docx]

Supplementary Materials

Supplementary table1. Sequence data of primers and taqman probes and the amplification data

| Gene Symbol | GenBank accession no | Strand | Nucleotide sequence | Location | Reporter | Quencher | Size of PCR products (bp) | Concentration in amplification MIX | Amplification efficiencies | |
| --- | --- | --- | --- | --- | --- | --- | --- | --- | --- | --- |
| RPL13 | NM_031101.1 | Sense | TCGTGAGGTGCCCTACAGTTAG | 209-230 |  |  | 107 | 0.050μM | | 101.5% |
|  |  | Probe | CACACCAAGGTCCGGGCTGGCAG | 235-257 | FAM | BQH |  | 0.050μM | |  |
|  |  | Antisense | GGTGCGTGCCATTTTCTTGTG | 315-295 |  |  |  | 0.050μM | |  |
| RPL32 | NM_013226.2 | Sense | ATCTGGCCCTTGAATCTTCTCC | 99-121 |  |  | 115 | 0.050μM | | 100.3% |
|  |  | Probe | TGTCGATGCCTCTGGGTTTCCGCC | 182-159 | Cy5 | BQH |  | 0.025μM | |  |
|  |  | Antisense | AGAGGACCAAGAAGTTCATCAGG | 213-192 |  |  |  | 0.050μM | |  |
| Rae1 | NM_001033708 | Sense | AAGCTGAAGACCTCAGAGCAG | 1024-1044 |  |  | 119 | 0.072μM | | 101.6% |
|  |  | Probe | CCGTGGCAGCGTGTGGCTTCAACC | 1055-1078 | ROX | BQH |  | 0.072μM | |  |
|  |  | Antisense | TTATAAAACTCATGCCCCTTGGAC | 1142-1119 |  |  |  | 0.072μM | |  |
| Myg1 | NM_001005545 | Sense | ACCTCGCAACAACCTCATGG | 109-128 |  |  | 133 | 0.084μM | | 94.0% |
|  |  | Probe | CGAATCGGGACGCACAACGGCAC | 137-159 | HEX | BQH |  | 0.098μM | |  |
|  |  | Antisense | CCGAGTCCGCACAATCTCTG | 241-222 |  |  |  | 0.084μM | |  |
| Rabepk | NM_001024871 | Sense | AAGCTGCATGGTACACTCTGAC | 475-496 |  |  | 119 | 0.098μM | | 99.0% |
|  |  | Probe | TGCCCTGGAGACAAGCCCTGCCC | 498-520 | HEX | BQH |  | 0.098μM | |  |
|  |  | Antisense | CCCGACAATGAAGACCTTCCC | 593-573 |  |  |  | 0.098μM | |  |
| Tmem100 | NM_001017479 | Sense | TCAAGTGGCTTCTCTGGTAATGG | 122-144 |  |  | 88 | 0.098μM | | 95.3% |
|  |  | Probe | TGTGAGCTTCCACCCTGGTCAGGC | 155-178 | ROX | BQH |  | 0.084μM | |  |
|  |  | Antisense | CTTATCTCGCTTCACAGGCTAGG | 209-187 |  |  |  | 0.098μM | |  |
| Rcc1l | NM_001108332 | Sense | GAGCACACTCCCTTGTCCTG | 592-611 |  |  | 113 | 0.084μM | | 100.1% |
|  |  | Probe | CCACCACCTTCCGTCCACACTGCC | 678-655 | HEX | BQH |  | 0.084μM | |  |
|  |  | Antisense | GTGACTCTCACTGTATACTTCGTC | 704-681 |  |  |  | 0.084μM | |  |
| Rhbdd3 | NM_001013875 | Sense | AGCTGAATCCGACCCAGACC | 159-178 |  |  | 127 | 0.112μM | | 97.3% |
|  |  | Probe | ACTCCCCTCTCTGTGCTCCACCCG | 184-207 | HEX | BQH |  | 0.112μM | |  |
|  |  | Antisense | CGGTAACACTCCTCCAACCATATC | 285-262 |  |  |  | 0.112μM | |  |
| Trit1 | NM_001108676 | Sense | GCCCTCTCAAGTTCCCTAACC | 705-725 |  |  | 127 | 0.080μM | | 99.5% |
|  |  | Probe | CGGCAGCAAGCATGTCGTCCACTC | 800-777 | HEX | BQH |  | 0.112μM | |  |
|  |  | Antisense | CTGTGAAAATCTCTCAGCTCTTCC | 831-808 |  |  |  | 0.080μM | |  |
| Prrx2 | NM_001105739 | Sense | GGCCAAAGAGTTCAGCTTACAC | 853-874 |  |  | 108 | 0.080μM | | 101.1% |
|  |  | Probe | ACAGCCAGGTGCCCACAGTGAACT | 876-899 | HEX | BQH |  | 0.112μM | |  |
|  |  | Antisense | ACTTCCTTTACTGCGGTCTGC | 960-940 |  |  |  | 0.080μM | |  |
| Lrrc41 | NM_001009710 | Sense | CACGAAAGCCTTCTAACCATTCC | 1271-1293 |  |  | 135 | 0.050μM | | 100.7% |
|  |  | Probe | AGCACGGAGCCCTCGAAGAAGCCC | 1380-1357 | HEX | BQH |  | 0.060μM | |  |
|  |  | Antisense | CATCCATGTCAGCGGCTCTG | 1405-1386 |  |  |  | 0.050μM | |  |
| Abhd2 | NM_001106275 | Sense | CACGAAAGCCTTCTAACCATTCC | 1271-1293 |  |  | 135 | 0.050μM | | 97.3% |
|  |  | Probe | AGCACGGAGCCCTCGAAGAAGCCC | 1380-1357 | HEX | BQH |  | 0.060μM | |  |
|  |  | Antisense | CATCCATGTCAGCGGCTCTG | 1405-1386 |  |  |  | 0.050μM | |  |
| Prr5 | NM_001012121 | Sense | CCTGTACCCATTCCTGTATCCTTG | 849-872 |  |  | 147 | 0.096μM | | 98.4% |
|  |  | Probe | TCCTACGCCGCTCCCGCTCTGG | 882-903 | ROX | BQH |  | 0.096μM | |  |
|  |  | Antisense | CTGTGCCTTCTGCCTCATGC | 995-976 |  |  |  | 0.096μM | |  |
| Fbxw4 | NM_001107600 | Sense | CTACTGTCCTGTGGTTATGATACC | 1071-1094 |  |  | 127 | 0.072μM | | 101.0% |
|  |  | Probe | CCGCTACTGGGACCTCCGCACAAG | 1100-1123 | HEX | BQH |  | 0.072μM | |  |
|  |  | Antisense | GGTTGCCGTCTGTCTGTAGG | 1197-1178 |  |  |  | 0.072μM | |  |
| Ipo4 | NM_001106038 | Sense | CCCTCAGATCCGCCAGTTTG | 221-240 |  |  | 111 | 0.060μM | | 101.5% |
|  |  | Probe | CTCTCCCGCTGCTCAGGTGCCAAG | 307-284 | ROX | BQH |  | 0.084μM | |  |
|  |  | Antisense | GCTGTCAGGACCAGAGACTTG | 331-311 |  |  |  | 0.060μM | |  |
| Lin37 | NM_001106245 | Sense | TGGTACCCAGACTTACCTTTGAC | 65-87 |  |  | 150 | 0.098μM | | 96% |
|  |  | Probe | CCTGCTCGCCTTCCGCCTCGC | 93-113 | ROX | BQH |  | 0.098μM | |  |
|  |  | Antisense | TCCATCTCTGATTTCTCCACTTTC | 214-191 |  |  |  | 0.098μM | |  |
| Prr3 | NM_212544 | Sense | CCGAAACGAAAGAAGCAGAACC | 43-64 |  |  | 150 | 0.112μM | | 101.2% |
|  |  | Probe | AGCCGCCGCAGCATCTCGCAC | 71-91 | HEX | BQH |  | 0.098μM | |  |
|  |  | Antisense | ATCACCAGGCTTTCCGTTAGC | 192-172 |  |  |  | 0.112μM | |  |
| Arid5a | NM_001034934 | Sense | ACCAAGCCCAGGAAGCAATAC | 259-279 |  |  | 140 | 0.072μM | | 96.2% |
|  |  | Probe | TGTGGTCTGGTCCGCCTGCCTCTC | 372-349 | HEX | BQH |  | 0.084μM | |  |
|  |  | Antisense | GGGGCATCTGATTTGGTCTTTTC | 398-376 |  |  |  | 0.072μM | |  |
| Sc65 | NM_021581 | Sense | GGAGATGAGTCCCTCACTGATC | 618-639 |  |  | 144 | 0.096μM | | 98.6% |
|  |  | Probe | CCGCTCCATGTGTTCTGTGCTGCT | 731-708 | ROX | BQH |  | 0.096μM | |  |
|  |  | Antisense | AGCAAAGACGGTCATATAATCAGC | 761-738 |  |  |  | 0.096μM | |  |
| Tmem45b | NM_001033067 | Sense | CCTTGTTTACTGCTTTCTGACTCG | 943-966 |  |  | 150 | 0.072μM | | 99.2% |
|  |  | Probe | AGGTCACTCCTCATCCGAGCCGCT | 1069-1046 | ROX | BQH |  | 0.072μM | |  |
|  |  | Antisense | AGGTCTCCACAGCTCCAAGC | 1092-1073 |  |  |  | 0.072μM | |  |
| Leprot | NM_020099 | Sense | GGGATTGTTGTTTCTGCCTTTGG | 245-267 |  |  | 120 | 0.060μM | | 98.0% |
|  |  | Probe | TGCCAGCCAGCACAAGACCACAGG | 335-312 | HEX | BQH |  | 0.072μM | |  |
|  |  | Antisense | GCCTTGGATCGTGAGGAAAATAAC | 364-341 |  |  |  | 0.060μM | |  |
| Fam210a | NM_001007688 | Sense | ACGGCCTACGCCATGTTTAAG | 723-743 |  |  | 100 | 0.084μM | | 96.9% |
|  |  | Probe | CGCCACGCCTGCCCGCTACA | 746-765 | HEX | BQH |  | 0.084μM | |  |
|  |  | Antisense | TGTAGCCATGACTTCGCAGATAC | 822-800 |  |  |  | 0.084μM | |  |
| Impact | NM_001012235 | Sense | AAGGTTCTTGCCAAGTTGTATGAG | 681-704 |  |  | 128 | 0.098μM | | 94.3% |
|  |  | Probe | TCGCCAGTGCCACCCACAACATCT | 715-738 | HEX | BQH |  | 0.098μM | |  |
|  |  | Antisense | GCTGTTTCTCCATCATCTTCGG | 808-787 |  |  |  | 0.098μM | |  |
| Asb5 | NM_001044247 | Sense | GGTCGTCTTCTTGCTCTGAGG | 307-327 |  |  | 138 | 0.160μM | | 99.5% |
|  |  | Probe | CCACATGGTCACCCAGGCAGGCTT | 415-392 | HEX | BQH |  | 0.128μM | |  |
|  |  | Antisense | TCCAGCTTCCAGGAGAGTCC | 444-425 |  |  |  | 0.160μM | |  |
| Hs6st1 | NM_001108210 | Sense | TCGCCCAGAAAGTTCTACTACATC | 336-359 |  |  | 112 | 0.060μM | |  |
|  |  | Probe | CCTGCTGCGAGACCCTGTATCCCG | 362-385 | ROX | BQH |  | 0.072μM | | 94.2% |
|  |  | Antisense | ACATGTGCAAGGAGGTCTTCC | 447-427 |  |  |  | 0.060μM | |  |
| Samd4b | NM_001107498 | Sense | GTACCCAAGCCCGCTTTCTG | 311-330 |  |  | 133 | 0.080μM | | 99.6% |
|  |  | Probe | CCTGGAGCACTCACTGGCGGACTG | 339-362 | ROX | BQH |  | 0.112μM | |  |
|  |  | Antisense | ACCTTCTCTTTGGACTCCTGTTG | 443-421 |  |  |  | 0.080μM | |  |
| Dclre1b | NM_001025687 | Sense | TATCCCACATGCACTGTGACC | 287-307 |  |  | 147 | 0.070μM | | 94.0% |
|  |  | Probe | CCTGTCTAGCACTTGGGCACGGC | 318-340 | ROX | BQH |  | 0.098μM | |  |
|  |  | Antisense | GGCTCTCACCAATCTCCAGAG | 433-413 |  |  |  | 0.070μM | |  |
| Dennd5a | NM_001107546 | Sense | TACCATCCGTCAGCCCAAAC | 2424-2443 |  |  | 129 | 0.098μM | |  |
|  |  | Probe | CCTGTCTCCCTCGGTCATTGCCCA | 2451-2474 | ROX | BQH |  | 0.098μM | |  |
|  |  | Antisense | CCCATCTTCTCTACCAGCATCC | 2552-2531 |  |  |  | 0.098μM | |  |
| Ier3 | NM_212505 | Sense | CGTGCGTCCGAACACTTCTC | 179-198 |  |  | 100 | 0.060μM | | 95.9% |
|  |  | Probe | CGAAAACGCAGCCGACGGGTGCTC | 207-230 | ROX | BQH |  | 0.084μM | |  |
|  |  | Antisense | AATGTTGGGTTCCTCGGTTGG | 278-258 |  |  |  | 0.060μM | |  |
| Slfn3/4 | NM_053687 | Sense | AAAGGCCCTCTTCAGTCAAGC | 2567-2587 |  |  | 150 | 0.060μM | | 98.7% |
|  |  | Probe | CTGCCACACAGTCCCCGTAGCTGC | 2591-2614 | HEX | BQH |  | 0.084μM | |  |
|  |  | Antisense | TGAGAACAGTTTCCCGCAGAG | 2716-2696 |  |  |  | 0.060μM | |  |

Supplementary table 2. The expression level and standard deviation of mRNAs

| Wound Age | Slfn3/4 | Abhd2 | Ier3 | Rhbdd3 | Tmem100 | Sc65 | Rabepk |
| --- | --- | --- | --- | --- | --- | --- | --- |
| C | 1±1.076 | 1±0.468 | 1±0.429 | 1±0.350 | 1±0.274 | 1±0.435 | 1±0.694 |
| 4H | 2.373±2.236 | 9.849±3.311* | 3.202±2.091* | 1.716±0.517 | 1.208±0.454 | 1.395±0.728 | 0.929±0.350 |
| 8H | 1.878±1.598 | 11.865±6.184* | 2.309±1.091 | 1.240±0.329 | 1.495±0.650 | 1.190±0.495 | 0.752±0.486 |
| 12H | 1.361±0.782 | 9.515±2.001* | 1.676±0.724 | 1.126±0.413 | 1.146±0.749 | 1.140±0.483 | 0.416±0.436 |
| 16H | 13.397±4.759* | 13.669±7.586* | 3.679±1.819* | 1.126±0.550* | 1.087±0.599 | 1.742±0.524* | 0.862±0.435 |
| 20H | 4.064±3.401 | 8.065±1.421* | 3.399±2.226* | 0.934±0.337 | 0.553±0.175 | 0.605±0.270 | 0.446±0.438 |
| 24H | 5.678±2.954* | 12.024±4.276* | 3.767±1.716* | 2.677±0.843* | 1.564±0.941 | 2.126±0.634* | 0.975±0.515 |
| 28H | 1.904±2.435 | 7.280±2.381* | 2.916±0.899* | 2.529±1.203* | 0.554±0.185 | 0.963±0.559 | 2.107±0.360* |
| 32H | 0.662±0.416 | 8.030±4.459* | 2.522±0.684* | 1.397±0.650 | 1.002±0.635 | 0.661±0.195 | 0.643±0.419 |
| 36H | 1.242±0.813 | 12.091±3.501* | 1.730±0.859 | 2.662±0.870* | 1.692±1.229 | 1.817±0.468* | 1.218±0.592 |
| 40H | 1.649±1.422 | 8.491±4.844* | 1.995±0.526 | 1.578±0.848 | 1.343±0.875 | 1.315±0.423 | 0.864±0.648 |
| 44H | 1.794±1.059 | 13.780±5.747* | 2.980±1.564* | 2.525±0.508* | 1.879±0.951* | 1.531±0.823* | 1.399±0.872 |
| 48H | 1.928±1.051 | 13.152±3.563* | 2.045±1.028 | 1.738±0.395 | 1.179±0.366 | 0.974±0.534 | 1.036±0.372 |
| Wound Age | Leprot | Trit1 | Rcc1l | Tmem45b | Samd4b | Hs6st1 | Prr5 |
| C | 1±0.420 | 1±0.396 | 1±0.445 | 1±0.720 | 1±0.430 | 1±0.546 | 1±0.355 |
| 4H | 0.707±0.326 | 0.839±0.305 | 3.043±0.716* | 0.404±0.222* | 1.337±0.931 | 0.920±0.646 | 2.162±1.009* |
| 8H | 0.833±0.506 | 0.945±0.516 | 2.461±0.837* | 0.553±0.359 | 1.252±0.682 | 1.368±0.984 | 1.113±0.488 |
| 12H | 0.651±0.247 | 0.938±0.374 | 1.410±0.469 | 0.926±0.524 | 0.957±0.666 | 0.809±0.540 | 0.995±0.474 |
| 16H | 0.953±0.202 | 1.159±0.383 | 1.910±0.574* | 0.702±0.483 | 2.524±0.719* | 1.892±0.784* | 1.949±0.526* |
| 20H | 1.248±0.348 | 1.358±0.576 | 1.576±0.301 | 0.329±0.494* | 2.071±0.542* | 1.401±0.695 | 1.502±0.778 |
| 24H | 1.616±0.740* | 1.317±0.370 | 2.316±0.701* | 0.385±0.224* | 2.017±1.208* | 1.391±0.836 | 1.472±0.821 |
| 28H | 1.483±0.341* | 1.673±0.515* | 1.411±0.279 | 0.582±0.372 | 2.015±0.428* | 2.049±0.631* | 1.475±0.424 |
| 32H | 1.703±0.498* | 1.416±0.488 | 1.561±0.554 | 1.150±0.484 | 1.547±0.564 | 1.325±0.496 | 1.439±0.392 |
| 36H | 1.149±0.743 | 1.389±0.628 | 2.104±0.569* | 1.395±0.675* | 1.282±1.068 | 1.116±0.834 | 1.073±0.553 |
| 40H | 1.497±0.537* | 1.365±0.463 | 2.063±0.741* | 1.274±0.582 | 1.451±0.731 | 1.391±0.777 | 1.249±0.549 |
| 44H | 1.591±0.457* | 1.283±0.371 | 2.037±0.448* | 0.863±0.381 | 1.763±0.717 | 1.817±0.640* | 1.495±0.753 |
| 48H | 1.454±0.532* | 1.306±0.386 | 1.398±0.246 | 0.915±0.374 | 1.471±0.658 | 1.202±0.710 | 1.449±0.687 |
| Wound Age | Arid5a | Ipo4 | Prr3 | Rae1 | Myg1 | Prrx2 | Lrrc41 |
| C | 1±0.451 | 1±0.327 | 1±0.501 | 1±0.253 | 1±0.203 | 1±0.226 | 1±0.279 |
| 4H | 1.739±1.076 | 1.422±0.701 | 1.377±0.332 | 0.929±0.265 | 0.893±0.265 | 0.182±0.089* | 0.674±0.227* |
| 8H | 1.935±0.955 | 0.885±0.511 | 1.827±0.665* | 1.198±0.260 | 0.802±0.378 | 0.628±0.205* | 0.750±0.191* |
| 12H | 1.574±0.965 | 1.448±0.621 | 0.919±0.504 | 0.951±0.329 | 0.540±0.140* | 0.761±0.338 | 0.398±0.151* |
| 16H | 4.677±1.188* | 2.352±0.511* | 1.409±0.641 | 1.648±0.230* | 0.712±0.158* | 0.801±0.379 | 0.545±0.106* |
| 20H | 3.321±1.566* | 0.913±0.416 | 0.768±0.372 | 1.411±0.228* | 0.578±0.127* | 0.671±0.179* | 0.277±0.055* |
| 24H | 2.555±0.989* | 2.982±1.303* | 0.882±0.301 | 1.607±0.314* | 0.739±0.105* | 1.043±0.411 | 0.459±0.134* |
| 28H | 1.764±0.405 | 1.127±0.273 | 1.783±0.612* | 1.050±0.330 | 0.693±0.223* | 0.556±0.277* | 0.586±0.112* |
| 32H | 1.188±0.336 | 0.601±0.248 | 0.809±0.379 | 0.719±0.222* | 0.684±0.140* | 0.628±0.154* | 0.304±0.111* |
| 36H | 0.778±0.533 | 1.455±0.354 | 1.191±0.256 | 0.918±0.308 | 0.905±0.111 | 0.806±0.297 | 0.466±0.087* |
| 40H | 1.666±0.639 | 2.624±1.032* | 2.168±0.498* | 1.060±0.200 | 0.833±0.266 | 0.571±0.238* | 0.574±0.071* |
| 44H | 1.533±0.635 | 2.614±0.985* | 1.168±0.311 | 1.115±0.221 | 0.883±0.108 | 0.640±0.144* | 0.503±0.095* |
| 48H | 2.064±0.917* | 1.252±0.357 | 1.224±0.190 | 1.091±0.377 | 0.804±0.137 | 0.942±0.546 | 0.420±0.064* |
| Wound Age | Lin37 | Fam210a | Impact | Asb5 | Dclre1b | Dennd5a | Fbxw4 |
| C | 1±0.249 | 1±0.373 | 1±0.344 | 1±0.077 | 1±0.404 | 1±0.410 | 1±0.225 |
| 4H | 0.913±0.170 | 0.887±0.366 | 0.816±0.202 | 1.123±0.487 | 1.426±0.249* | 1.122±0.309 | 0.687±0.212 |
| 8H | 0.912±0.249 | 0.899±0.324 | 0.811±0.158 | 1.191±0.694 | 1.420±0.345* | 0.780±0.228 | 0.829±0.575 |
| 12H | 0.584±0.105* | 0.898±0.229 | 0.600±0.186* | 1.411±0.462 | 0.819±0.356 | 0.441±0.185* | 0.483±0.235* |
| 16H | 1.126±0.298 | 0.605±0.251* | 0.705±0.218* | 1.379±0.820 | 1.287±0.226 | 0.584±0.174* | 0.789±0.140 |
| 20H | 0.797±0.213 | 0.679±0.233* | 0.436±0.096* | 1.780±0.696* | 0.882±0.234 | 0.397±0.154* | 0.945±0.266 |
| 24H | 1.324±0.380* | 0.565±0.127* | 0.783±0.262 | 1.228±0.224 | 1.148±0.311 | 0.681±0.205* | 0.859±0.501 |
| 28H | 1.119±0.344 | 0.627±0.175* | 1.461±0.733* | 1.267±0.376 | 1.761±0.316* | 1.275±0.288* | 0.638±0.203 |
| 32H | 0.919±0.283 | 0.465±0.135* | 0.482±0.188* | 0.989±0.164 | 1.021±0.288 | 0.503±0.214* | 0.600±0.161* |
| 36H | 1.332±0.295* | 0.589±0.255* | 0.535±0.096* | 0.854±0.090 | 1.253±0.233 | 0.791±0.200 | 0.800±0.506 |
| 40H | 1.060±0.374 | 0.732±0.303* | 0.750±0.301 | 0.732±0.099 | 1.374±0.371* | 0.886±0.264 | 1.089±0.275 |
| 44H | 1.203±0.185 | 0.727±0.163* | 0.471±0.067* | 0.786±0.290 | 1.538±0.419* | 0.888±0.132 | 1.037±0.266 |
| 48H | 0.965±0.088 | 0.606±0.254* | 0.489±0.073* | 0.572±0.145* | 1.407±0.237* | 0.831±0.194 | 0.870±0.488 |

“*”represents a significant difference between the injury group and the control group (P<0.05)

Supplementary table 3. The CV scores of all indicators

| AREs | GO | mRNA | C | 4H | 8H | 12H | 16H | 20H | 24H | 28H | 32H | 36H | 40H | 44H | 48H | Sum of | ARE | GO | Subgroup |
| --- | --- | --- | --- | --- | --- | --- | --- | --- | --- | --- | --- | --- | --- | --- | --- | --- | --- | --- | --- |
|  |  |  |  |  |  |  |  |  |  |  |  |  |  |  |  | Scores |  |  |  |
| - | CC | Rae1 | 4 | 4 | 4 | 3 | 4 | 4 | 4 | 2 | 3 | 3 | 4 | 4 | 3 | 46 | 34.88 | 34.5 | 38.33 |
| - | CC | Myg1 | 4 | 3 | 3 | 4 | 4 | 4 | 4 | 2 | 4 | 4 | 3 | 4 | 4 | 47 |  |  |  |
| - | CC | Rabepk | 1 | 2 | 1 | 1 | 1 | 1 | 1 | 4 | 1 | 2 | 1 | 1 | 3 | 20 |  |  |  |
| - | CC | Tmem100 | 4 | 3 | 3 | 1 | 1 | 3 | 1 | 2 | 1 | 1 | 1 | 1 | 3 | 25 |  |  |  |
| - | CC | Lin37 | 4 | 4 | 4 | 4 | 3 | 3 | 3 | 3 | 3 | 4 | 3 | 4 | 4 | 46 |  |  |  |
| - | CC | Lrrc41 | 3 | 3 | 4 | 3 | 4 | 4 | 3 | 4 | 2 | 4 | 4 | 4 | 4 | 46 |  |  |  |
| - | BP | Prrx2 | 4 | 2 | 4 | 2 | 2 | 3 | 2 | 1 | 4 | 2 | 2 | 3 | 1 | 32 |  | 31.44 | 31.8 |
| - | BP | Abhd2 | 1 | 3 | 2 | 4 | 1 | 4 | 2 | 2 | 1 | 3 | 1 | 2 | 3 | 29 |  |  |  |
| - | BP | Prr5 | 3 | 2 | 3 | 2 | 3 | 1 | 1 | 3 | 4 | 2 | 2 | 1 | 1 | 28 |  |  |  |
| - | BP | Fbxw4 | 4 | 3 | 1 | 2 | 4 | 3 | 1 | 2 | 4 | 2 | 4 | 3 | 1 | 34 |  |  |  |
| - | BP | Rhbdd3 | 3 | 3 | 4 | 3 | 3 | 3 | 3 | 1 | 1 | 3 | 1 | 4 | 4 | 36 |  |  |  |
| - | MF | Ipo4 | 3 | 2 | 2 | 2 | 4 | 2 | 2 | 3 | 2 | 3 | 2 | 2 | 2 | 31 |  | 32.67 | 33.8 |
| - | MF | Prr3 | 1 | 4 | 3 | 1 | 2 | 2 | 3 | 1 | 1 | 4 | 4 | 3 | 4 | 33 |  |  |  |
| - | MF | Arid5a | 1 | 1 | 2 | 1 | 4 | 2 | 2 | 4 | 3 | 1 | 2 | 2 | 2 | 27 |  |  |  |
| - | MF | Trit1 | 3 | 3 | 2 | 3 | 2 | 2 | 4 | 2 | 3 | 2 | 3 | 3 | 3 | 35 |  |  |  |
| - | MF | Rcc1l | 2 | 4 | 3 | 4 | 3 | 4 | 3 | 4 | 2 | 3 | 3 | 4 | 4 | 43 |  |  |  |
| + | CC | Sc65 | 2 | 1 | 3 | 3 | 3 | 2 | 3 | 1 | 3 | 3 | 3 | 1 | 1 | 29 | 30.33 | 34.5 | 28.75 |
| + | CC | Tmem45b | 1 | 1 | 1 | 1 | 1 | 1 | 1 | 1 | 2 | 2 | 2 | 1 | 2 | 17 |  |  |  |
| + | CC | Leprot | 2 | 2 | 1 | 3 | 4 | 3 | 2 | 4 | 3 | 1 | 3 | 3 | 2 | 33 |  |  |  |
| + | CC | Fam210a | 3 | 2 | 3 | 4 | 2 | 3 | 4 | 3 | 3 | 2 | 2 | 3 | 2 | 36 |  |  |  |
| + | BP | Impact | 3 | 4 | 4 | 4 | 3 | 4 | 3 | 1 | 2 | 4 | 2 | 4 | 4 | 42 |  | 31.44 | 31 |
| + | BP | Asb5 | 4 | 2 | 1 | 4 | 1 | 2 | 4 | 3 | 4 | 4 | 4 | 2 | 3 | 38 |  |  |  |
| + | BP | Hs6st1 | 1 | 1 | 1 | 1 | 2 | 2 | 1 | 3 | 2 | 1 | 1 | 2 | 1 | 19 |  |  |  |
| + | BP | Ier3 | 2 | 1 | 3 | 2 | 1 | 1 | 2 | 2 | 4 | 1 | 4 | 1 | 1 | 25 |  |  |  |
| + | MF | Samd4b | 2 | 1 | 2 | 1 | 3 | 4 | 1 | 4 | 2 | 1 | 1 | 2 | 2 | 26 |  | 32.67 | 31.25 |
| + | MF | Dclre1b | 2 | 4 | 4 | 2 | 4 | 4 | 4 | 4 | 3 | 4 | 4 | 3 | 4 | 46 |  |  |  |
| + | MF | Dennd5a | 2 | 4 | 4 | 3 | 3 | 2 | 3 | 4 | 1 | 3 | 3 | 4 | 3 | 39 |  |  |  |
| + | MF | Slfn3/4 | 1 | 1 | 1 | 1 | 1 | 1 | 2 | 1 | 1 | 1 | 1 | 1 | 1 | 14 |  |  |  |

Supplementary table 4. The CV’CV of all indicators

| AREs | GO | mRNA | C | 4H | 8H | 12H | 16H | 20H | 24H | 28H | 32H | 36H | 40H | 44H | 48H | CV’CV | ARE | GO | Subgroup |
| --- | --- | --- | --- | --- | --- | --- | --- | --- | --- | --- | --- | --- | --- | --- | --- | --- | --- | --- | --- |
| - | CC | Rae1 | 0.253 | 0.286 | 0.217 | 0.346 | 0.140 | 0.161 | 0.195 | 0.314 | 0.309 | 0.336 | 0.189 | 0.198 | 0.346 | 0.291 | 0.321 | 0.337 | 0.345 |
| - | CC | Myg1 | 0.203 | 0.297 | 0.472 | 0.259 | 0.222 | 0.221 | 0.142 | 0.322 | 0.205 | 0.123 | 0.319 | 0.122 | 0.171 | 0.414 |  |  |  |
| - | CC | Rabepk | 0.694 | 0.377 | 0.646 | 1.047 | 0.505 | 0.981 | 0.528 | 0.171 | 0.651 | 0.486 | 0.750 | 0.624 | 0.359 | 0.402 |  |  |  |
| - | CC | Tmem100 | 0.274 | 0.375 | 0.435 | 0.653 | 0.551 | 0.316 | 0.602 | 0.333 | 0.634 | 0.726 | 0.651 | 0.506 | 0.311 | 0.320 |  |  |  |
| - | CC | Lin37 | 0.249 | 0.186 | 0.273 | 0.179 | 0.265 | 0.267 | 0.287 | 0.307 | 0.308 | 0.221 | 0.353 | 0.154 | 0.092 | 0.300 |  |  |  |
| - | CC | Lrrc41 | 0.279 | 0.336 | 0.254 | 0.379 | 0.194 | 0.200 | 0.292 | 0.191 | 0.367 | 0.188 | 0.124 | 0.188 | 0.151 | 0.342 |  |  |  |
| - | BP | Prrx2 | 0.226 | 0.490 | 0.326 | 0.445 | 0.474 | 0.267 | 0.394 | 0.499 | 0.246 | 0.369 | 0.417 | 0.225 | 0.580 | 0.305 |  | 0.345 | 0.325 |
| - | BP | Abhd2 | 0.468 | 0.336 | 0.521 | 0.210 | 0.555 | 0.176 | 0.356 | 0.327 | 0.555 | 0.290 | 0.571 | 0.417 | 0.271 | 0.350 |  |  |  |
| - | BP | Prr5 | 0.355 | 0.467 | 0.439 | 0.477 | 0.270 | 0.518 | 0.558 | 0.288 | 0.273 | 0.515 | 0.440 | 0.504 | 0.474 | 0.232 |  |  |  |
| - | BP | Fbxw4 | 0.225 | 0.309 | 0.693 | 0.486 | 0.177 | 0.281 | 0.584 | 0.318 | 0.268 | 0.632 | 0.253 | 0.257 | 0.561 | 0.455 |  |  |  |
| - | BP | Rhbdd3 | 0.350 | 0.301 | 0.265 | 0.367 | 0.297 | 0.360 | 0.315 | 0.476 | 0.465 | 0.327 | 0.538 | 0.201 | 0.228 | 0.284 |  |  |  |
| - | MF | Ipo4 | 0.327 | 0.493 | 0.578 | 0.429 | 0.217 | 0.456 | 0.437 | 0.242 | 0.413 | 0.243 | 0.393 | 0.377 | 0.417 | 0.272 |  | 0.304 | 0.287 |
| - | MF | Prr3 | 0.501 | 0.241 | 0.364 | 0.549 | 0.455 | 0.484 | 0.341 | 0.343 | 0.469 | 0.215 | 0.230 | 0.267 | 0.155 | 0.359 |  |  |  |
| - | MF | Arid5a | 0.451 | 0.619 | 0.493 | 0.613 | 0.254 | 0.471 | 0.387 | 0.230 | 0.283 | 0.686 | 0.384 | 0.414 | 0.444 | 0.319 |  |  |  |
| - | MF | Trit1 | 0.396 | 0.363 | 0.546 | 0.399 | 0.331 | 0.424 | 0.281 | 0.308 | 0.344 | 0.452 | 0.339 | 0.289 | 0.295 | 0.207 |  |  |  |
| - | MF | Rcc1l | 0.445 | 0.235 | 0.340 | 0.333 | 0.300 | 0.191 | 0.303 | 0.198 | 0.355 | 0.271 | 0.359 | 0.220 | 0.176 | 0.280 |  |  |  |
| + | CC | Sc65 | 0.435 | 0.522 | 0.416 | 0.424 | 0.301 | 0.447 | 0.298 | 0.580 | 0.295 | 0.258 | 0.322 | 0.538 | 0.548 | 0.268 | 0.343 | 0.337 | 0.325 |
| + | CC | Tmem45b | 0.720 | 0.550 | 0.650 | 0.566 | 0.688 | 1.500 | 0.581 | 0.639 | 0.420 | 0.484 | 0.456 | 0.441 | 0.409 | 0.454 |  |  |  |
| + | CC | Leprot | 0.420 | 0.461 | 0.607 | 0.379 | 0.212 | 0.279 | 0.458 | 0.230 | 0.293 | 0.647 | 0.359 | 0.287 | 0.366 | 0.348 |  |  |  |
| + | CC | Fam210a | 0.373 | 0.413 | 0.361 | 0.255 | 0.414 | 0.343 | 0.226 | 0.279 | 0.291 | 0.434 | 0.414 | 0.224 | 0.419 | 0.228 |  |  |  |
| + | BP | Impact | 0.344 | 0.248 | 0.195 | 0.309 | 0.308 | 0.220 | 0.335 | 0.501 | 0.389 | 0.180 | 0.402 | 0.143 | 0.149 | 0.380 |  | 0.345 | 0.371 |
| + | BP | Asb5 | 0.077 | 0.434 | 0.582 | 0.328 | 0.594 | 0.391 | 0.183 | 0.297 | 0.166 | 0.105 | 0.136 | 0.369 | 0.253 | 0.564 |  |  |  |
| + | BP | Hs6st1 | 0.546 | 0.702 | 0.720 | 0.667 | 0.414 | 0.496 | 0.601 | 0.308 | 0.374 | 0.747 | 0.559 | 0.352 | 0.591 | 0.270 |  |  |  |
| + | BP | Ier3 | 0.429 | 0.653 | 0.472 | 0.432 | 0.494 | 0.655 | 0.456 | 0.308 | 0.271 | 0.497 | 0.264 | 0.525 | 0.502 | 0.269 |  |  |  |
| + | MF | Samd4b | 0.430 | 0.696 | 0.545 | 0.696 | 0.285 | 0.262 | 0.599 | 0.213 | 0.364 | 0.833 | 0.504 | 0.407 | 0.448 | 0.385 |  | 0.304 | 0.331 |
| + | MF | Dclre1b | 0.404 | 0.175 | 0.243 | 0.434 | 0.175 | 0.265 | 0.271 | 0.180 | 0.282 | 0.186 | 0.270 | 0.273 | 0.169 | 0.332 |  |  |  |
| + | MF | Dennd5a | 0.410 | 0.275 | 0.292 | 0.420 | 0.298 | 0.387 | 0.300 | 0.226 | 0.425 | 0.253 | 0.299 | 0.149 | 0.234 | 0.276 |  |  |  |
| + | MF | Slfn3/4 | 1.076 | 0.942 | 0.851 | 0.575 | 0.676 | 0.837 | 0.520 | 1.279 | 0.628 | 0.654 | 0.862 | 0.590 | 0.545 | 0.296 |  |  |  |
